# Severe aseptic orbital cellulitis with subtenon carboplatin for intraocular retinoblastoma

**DOI:** 10.4103/0301-4738.73714

**Published:** 2011

**Authors:** Parag K Shah, N Kalpana, V Narendran, Minu Ramakrishnan

**Affiliations:** Department of Pediatric Retina and Ocular Oncology, Aravind Eye Hospital and Postgraduate Institute of Ophthalmology, Coimbatore, Tamil Nadu, India

**Keywords:** Aseptic orbital cellulitis, retinoblastoma, subtenon carboplatin

## Abstract

Retinoblastoma is a rare intraocular tumor of childhood. Chemoreduction followed by laser or cryotherapy is the treatment of choice. Subtenon carboplatin injection is also an accepted treatment modality for vitreous seeds, along with systemic chemotherapy. Transient periocular edema, optic neuropathy and fibrosis of orbital tissues are the known side effects of subteneon carboplatin injection. We report a case of severe aseptic orbital cellulitis with necrosis and prolapse of the conjunctiva 48 h after the injection, which resolved well on only conservative management.

Retinoblastoma is the most common intraocular malignancy of childhood. There is a drastic change in its management over the past 10 years. The trend[1] is shifting away from enucleation and external beam radiotherapy to chemoreduction followed by focal tumor consolidation. However, treatment of vitreous seeds is still a challenge. Subtenon carboplatin is now an accepted treatment for the vitreous seeds for intraocular retinoblastoma along with systemic chemotherapy.[2,3] Transient periocular edema is a common side effect of this treatment. We report a case of severe aseptic orbital cellulitis with necrosis and prolapse of the conjunctiva following a subtenon carboplatin injection in a patient with intraocular retinoblastoma with vitreous seeds.

## Case Report

**Figure 1:**
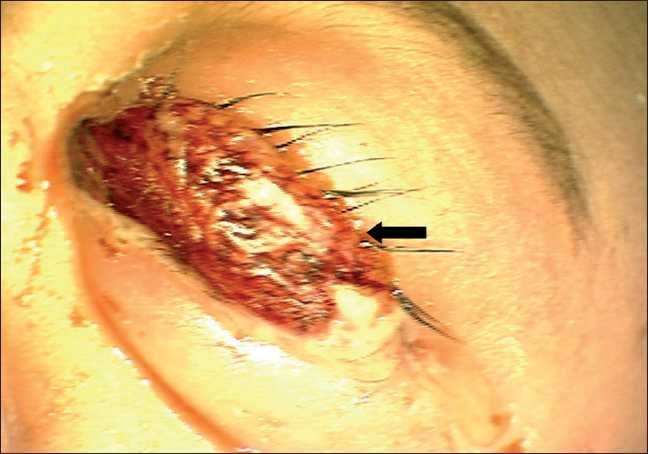
Left eye photograph showing lid edema with overhanging prolapsed necrotic conjunctiva (black arrow)

**Figure 2:**
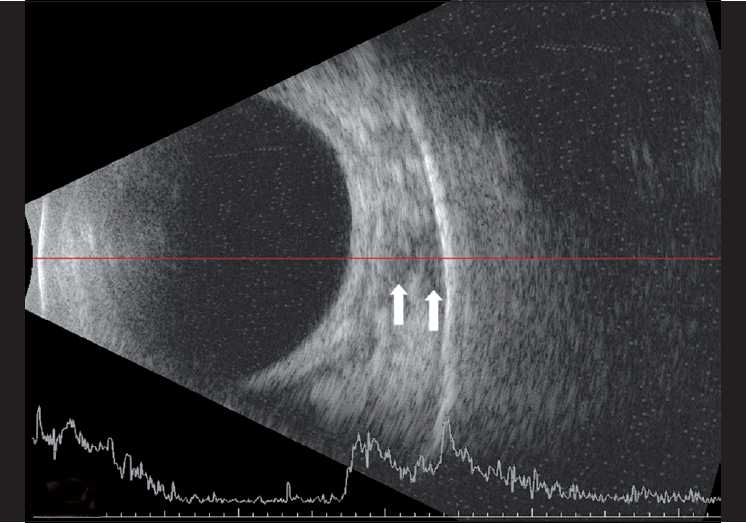
B-scan picture of the superior orbit showing fluid-filled pockets (white arrows)

**Figure 3:**
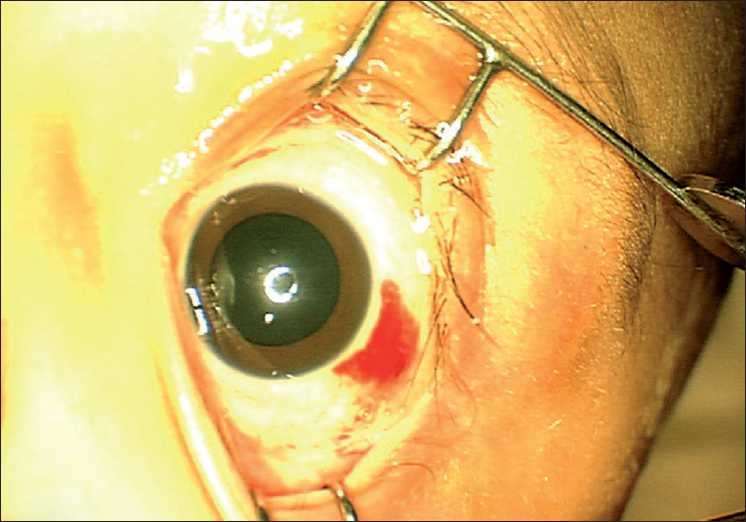
One-week follow-up photograph showing completely resolved lid edema with a small subconjunctival hemorrhage temporally

A 3-month-old female child presented to our clinic with parents complaining of white reflex in the right eye since 15 days. Examination under anesthesia (EUA) revealed circumcorneal congestion, corneal edema, shallow anterior chamber, and intraocular pressure of 54 mmHg on tonopen in the right eye (RE). Fundus showed a large yellowish mass lesion filling the whole vitreous cavity with calcification on B scan. The left eye (LE) anterior segment was normal. Fundus showed three small tumors (<1 disc diameter), one located just above the optic disc, one in the inferonasal quadrant and a third one in the superotemporal quadrant. Another large mass was seen in the nasal periphery with a height of 3.2 mm and a base of 6.1 mm with localized vitreous seeds suggestive of group C retinoblastoma. Transpupillary thermotherapy (TTT) was done immediately for the three small tumors in the LE. Magnetic resonance imaging (MRI) showed no extraocular extension. The child was started on systemic three-drug chemotherapy using vincristine, etoposide, and carboplatin and was planned to receive three subtenon injections of carboplatin in the LE a day before second, third, and fourth cycle. After the first cycle, the three small tumors in the LE had scarred while the large nasal mass had reduced in size with prominent persistent localized vitreous seeds. Two milliliters (20 mg) of subtenon carboplatin (Kemocarb, Dabur Pharma Ltd., Solan, India) was given using a 26 gauge blunt cannula after incising the conjunctiva and tenon’s capsule 8 mm posterior to the limbus in the inferotemporal quadrant. Due to cost factor, we reuse the 15-ml open vial of carboplatin for multiple patients across many days, under strict aseptic precautions. The child had mild periorbital swelling on the next day and was discharged for the second chemotherapy cycle. Three weeks later, just before the third cycle, the second dose of subtenon carboplatin was given in the same area. The child had lid edema and chemosis the next day and was discharged on topical steroids and antibiotic drops and oral paracetamol syrup. Two days later, the child presented with severe lid edema with overhanging prolapsed necrotic conjunctiva [Fig. 1]. There was mild proptosis with minimal restriction of extraocular movements. Rest of the anterior segment was normal. The child was afebrile. B-scan ultrasonography showed few echo-free pockets in the superior orbit, suggestive of fluid-filled spaces in the orbit [Fig. 2]. The child was started on empirical dexamethasone (0.5 ml twice daily) and cefotaxime (125 mg twice daily) systemically and necrotic conjunctiva was excised on the same day, with a temporary tarsorrhaphy for adequate lid closure. Necrosis was confined only to the prolapsed superior palpebral conjunctiva and was not extending posteriorly. Only this prolapsed necrotic part was excised and the remaining conjunctiva was anchored with the Pang suture using 6’0 vicryl. The Pang suture and central tarsorraphy was released after 3 days. The reused carboplatin vial was sent for culture which showed no growth. The patient recovered well in 3 days, and chemotherapy was resumed. One -week follow-up showed completely resolved lid edema with a small subconjunctival hemorrhage temporally [Fig. 3]. The ocular movements were normal. The outcome of the LE was good after completing the chemotherapy with flat scar formation over the tumors and a complete disappearance of the vitreous seeds. There was no complication or side effect seen in LE even after the 1-year follow-up. The RE was enucleated after six cycles of chemotherapy. The fundus showed a large partially calcified mass over the macula with a degenerated retina having multiple retinal folds and subretinal seeds.

## Discussion

Chemotherapy by itself cannot cure retinoblastoma, although it is efficient in reducing the tumor volume. Various chemoreduction protocols are used to shrink the tumor, so it can be amenable to treatment with cryotherapy, laser photocoagulation, thermotherapy, or plaque radiotherapy. In addition, subconjunctival carboplatin injections are now an accepted treatment for focal tumors[2,3] with vitreous seeds.

Various studies have been conducted to detect efficacy and toxicity of periocular carboplatin. It has been demonstrated that there is no toxic effect on the retinal function,[4] but optic neuropathy,[5] fibrosis of orbital soft tissues,[6] as well as aseptic preseptal cellulitis[7] have been reported. A histopathological study[5] on enucleated eyes with previous carboplatin injections demonstrated focal optic atrophy with loss of myelin sheaths and nerve fibers. These changes were attributed in part to the direct toxicity of carboplatin to the vascular endothelia of the pial vasculature. Also, the fibrovascular adipose tissue in close proximity to the injection site showed areas of necrosis, inflammation, and dystrophic calcification causing difficulty in enucleation if required.[6]

Aseptic or pseudo-preseptal cellulitis has been reported by Kiratli *et al*.[7] in a patient who received a subconjunctival injection of carboplatin. The patient developed pain and marked swelling of the eyelid 10 h after the procedure. The signs resolved rapidly even without medications.

Our patient developed aseptic orbital cellulitis following her second subtenon carboplatin injection. She presented with pain and lid edema with prolapsed necrotic conjunctiva 48 h after the procedure. She was treated empirically with systemic dexamethasone and cefotaxime. Prolapsed necrotic conjunctiva was excised, and the patient recovered well in 3 days. This is comparable to the study by Kiratli *et al*.,[7] which suggests that fat necrosis caused by carboplatin is believed to trigger an orbital inflammatory reaction. They have concluded that severe periocular noninfectious soft-tissue reaction following inferior subconjunctival carboplatin injection, while alarming, is self-limited and close observation is appropriate, as seen in our case.

Recent trials using a fibrin sealant[6,8,9] have shown controlled and localized release of carboplatin with good efficacy. This can be used in the future to limit the number of injections and prevent the dispersion of a large amount of drug into the orbital tissues, thereby avoiding most of the associated complications, including aseptic orbital cellulitis. We also recommend not to reuse the open carboplatin vial. We did not encounter this complication in other patients using a fresh vial every time.
